# Role of Pro-Inflammatory Cytokines and Biochemical Markers in the Pathogenesis of Type 1 Diabetes: Correlation with Age and Glycemic Condition in Diabetic Human Subjects

**DOI:** 10.1371/journal.pone.0161548

**Published:** 2016-08-30

**Authors:** Naureen Fatima, Syed Mohd Faisal, Swaleha Zubair, Mohd Ajmal, Sheelu Shafiq Siddiqui, Shagufta Moin, Mohammad Owais

**Affiliations:** 1 Department of Biochemistry, Jawaharlal Nehru Medical College, Aligarh Muslim University, Aligarh, Uttar Pradesh-202002, India; 2 Molecular Immunology Laboratory, Interdisciplinary Biotechnology Unit, Aligarh Muslim University, Aligarh, Uttar Pradesh-202002, India; 3 Women’s College, Aligarh Muslim University, Aligarh, Uttar Pradesh-202002, India; 4 Department of Anatomy, Jawaharlal Nehru Medical College, Aligarh Muslim University, Aligarh, Uttar Pradesh-202002, India; 5 Rajiv Gandhi Centre for Diabetes and Endocrinology, Aligarh Muslim University, Aligarh, Uttar Pradesh-202002, India; La Jolla Institute for Allergy and Immunology, UNITED STATES

## Abstract

**Background:**

Type 1 diabetes mellitus is a chronic inflammatory disease involving insulin producing β-cells destroyed by the conjoined action of auto reactive T-cells, inflammatory cytokines and monocytic cells. The aim of this study was to elucidate the status of pro-inflammatory cytokines and biochemical markers and possible correlation of these factors towards outcome of the disease.

**Methods:**

The study was carried out on 29 T1D subjects and 20 healthy subjects. Plasma levels of oxidative stress markers, enzymatic and non-enzymatic antioxidants were estimated employing biochemical assays. The levels of pro-inflammatory cytokines such as by IL-1β & IL-17 in the serum were determined by ELISA, while the expression of TNF-α, IL-23 & IFN-γ was ascertained by qRT-PCR.

**Results:**

The onset of T1D disease was accompanied with elevation in levels of Plasma malondialdehyde, protein carbonyl content and nitric oxide while plasma vitamin C, reduced glutathione and erythrocyte sulfhydryl groups were found to be significantly decreased in T1D patients as compared to healthy control subjects. Activity of antioxidant enzymes, superoxide dismutase, catalase, glutathione reductase and glutathione-s-transferase showed a significant suppression in the erythrocytes of T1D patients as compared to healthy subjects. Nevertheless, the levels of pro-inflammatory cytokines IL-1β and IL-17A were significantly augmented (***p≤.001) on one hand, while expression of T cell based cytokines IFN-γ, TNF-α and IL-23 was also up-regulated (*p≤.05) as compared to healthy human subjects.

**Conclusion:**

The level of pro-inflammatory cytokines and specific biochemical markers in the serum of the patient can be exploited as potential markers for type 1 diabetes pathogenesis. The study suggests that level of inflammatory markers is up-regulated in T1D patients in an age dependent manner.

## Introduction

Diabetes mellitus (DM) has been considered a pool of diverse metabolic disorders characterized by hyper-glycemic episodes due to relative or absolute insulin deficiency in the afflicted patients [1]. On the basis of etiology, DM has been categorized in two broad types viz., Type 1 and Type 2. Type 1 Diabetes (T1D) is an autoimmune pathology which results into selective elimination of insulin producing pancreatic β-cells owing to lymphocytic infiltration [2]. Previously known as insulin-dependent diabetes mellitus, this category of diabetes affects only 5–10% of total population in the world. The incidence of T1D has enormously increased in the past 40 years and increasingly affecting the individuals with genetic susceptibility [3, 4].

Several earlier studies have suggested involvement of both genetic and environmental factors in the pathogenesis of T1D. It is considered a polygenic disease whereby almost total genetic susceptibility lies within the human leukocyte antigen (HLA) [5]. Individuals with the HLA-risk genotypes DR3 and DR4 are more likely to develop the disease [6]. Several environmental factors involved in T1D pathogenesis account for infiltration of lymphocytesin the pancreatic islet tissues leading to insulitis which eventually results into β-cell destruction [7]. The infiltrating immune cells produce various pro-inflammatory cytokines which further exacerbate the T cell infiltration and apoptosis of β cells [7]. In general, T lymphocytes, dendritic cells, macrophages and various other immune cells are involved in β-cell destruction [8].

The hallmark of type 1 diabetes is diminutive insulin secretion that subsequently ensued in chronic hyperglycemia. Hyperglycemia inturn induces reactive oxygen species (ROS) production which overwhelms the antioxidant defence in the body leading to oxidative stress [9, 10]. The free radical generation leads to diabetes related complications and cause irreversible damage by altering proteins, carbohydrates and nucleic acids of the host cells. ROS cause injury to the β-cells of the pancreatic islets thereby activating the transcription factor NF-κβ in the macrophages which pose a greater threat of autoimmune attack to the islets [11–13]. In the present study, we have tried to elucidate the interplay between oxidative stress parameters and pro-inflammatory cytokines which are simultaneously responsible for the pathogenesis of T1D in children and adolescents below 25 years with varying duration of diabetes.

## Research Design and Methods

### Human subjects

T1D patients attending the OPD of Rajiv Gandhi Centre for Diabetes and Endocrinology, JNMCH, Aligarh, were selected for the study. The study group consisted of 29 patients with type 1 diabetes patients (12 females with mean age 14.25±4 years and 17 males with mean age 17.65±4.96 years) diagnosed according to the criteria provided by American Diabetes Association. Exclusion criteria were set as follows: autoimmune diseases (e.g. rheumatoid arthritis), steroid therapy and macrovascular complications (e.g. myocardial infarction) *etc*. All the patients were newly diagnosed who later started taking regular insulin injections after first blood withdrawal for further follow-up study. Healthy age matched individuals (n = 25) served as controls. The patients were grouped on the basis of two criteria: (i) according to age ≤15 years and >15 years, and (ii) according to their fasting blood sugar level ≤160 mg/dl and >160 mg/dl.

### Reagents

All reagents used in this study were of analytical grade. 5,5’-Dithiobis-2-Nitrobenzoic Acid (DTNB), 1-chloro-2,4-dinitrobenzene (CDNB), Thiobarbituric acid and Nicotinamide Adenine Dinucleotide Phosphate Dehydrogenase (NADPH) were purchased from Sisco Research Laboratories (India). 2,4,6-Tri-(2-Pyridyl)-5-Triazine (TPTZ), Pyragallol, Reduced Glutathione, Oxidized Glutathione, 2,4-Dinitrophenyl Hydrazine (DNPH), Tri-fluoroacetic Acid and Guanidine Hydrochloride were obtained from Sigma (USA). N-(1-Naphthyl) ethylene-diaminedihydrochloride (NED), Hydrogen Peroxide and Sulphanilamide were obtained from Merck (USA). PureLink@ RNA mini kit (Invitrogen), High Capacity cDNA Reverse Transcription Kit (Applied Biosystems), Primers for IFN-γ, TNF-α and β-actin procured and synthesized from Eurofins Genomics, India Pvt. Ltd., while ELISA cytokine detection kits procured from R&D Systems, Minneaopolis, USA. Highly purified distilled water was obtained from Millipore.

### Blood samples

Fasting blood (5 ml) was withdrawn from each patient and transferred into plain (clot activator vials) and anti-coagulant vials. Fresh whole blood was used for RT-PCR analysis. The plasma was obtained from whole blood while serum was separated from clotted blood by centrifugation at 1800g for 15min at 4°C. After removing plasma from the anticoagulant vials, buffy coat was removed and erythrocytes were washed thrice with isotonic saline. The erythrocytes were lysed with sodium phosphate buffer at 4°C. Plasma, sera and erythrocyte lysates were stored at -80°C until further experiments.

### Ethics Statement

The protocols, used in the study, were approved by the Institutional Ethics and Research Advisory Committee, Faculty of Medicine, Jawaharlal Nehru Medical College, Aligarh Muslim University, Aligarh-202002, India. The patients involved signed an informed consent prior to commencement of the study.

### Biomarkers of oxidative stress

The biomarkers of oxidative stress include protein carbonyls, nitric oxide and lipid peroxides like malondialdehyde. Protein oxidation, as assessed by the protein carbonyl contentin plasma was measured according to the method described by Levine [14]. Results were reported in nmol/mg protein. The amount of protein in the plasma samples were calculated by Lowry method [15]. The nitric oxide levels in the plasma samples were estimated by the method of Mathew *et al* [16]. Plasma Malondialdehyde (MDA) was estimated by the original protocol of Ohkawa *et al* [17].

### Total antioxidant capacity and erythrocyte total thiols

The antioxidant status is assessed by measuring the Ferric Reducing Ability of Plasma (FRAP) or Total Antioxidant Capacity by the published protocol of Benzie and Strain [18]. FRAP values are expressed in μmoles/L. Erythrocyte total thiols were determined according to Sedlak and Lindsay [19] and values were reported in nmol/mg haemoglobin. The haemoglobin content of erythrocyte lysate was estimated by Drabkin’s reagent using the cyanmet-haemoglobin method.

### Non-enzymatic antioxidants

The non-enzymatic antioxidants in the body include Vitamin C and Reduced Glutathione (GSH). Plasma Vitamin C level was measured by the method described by Kyaw [20]. A standard curve was prepared by using increasing concentrations of ascorbic acid for calculating the levels of Vitamin C. GSH, a tri-peptide of Glu-Cys-Gly, was estimated in the plasma by the method of Jollow *et al* [21] and the values were expressed in μmoles/ml.

### Assay of antioxidant enzymes

The activity of antioxidant enzymes viz. SOD, CAT, GR and GST were measured in the erythrocyte lysate and expressed in units/mg of haemoglobin. SOD was measured by the method involving auto-oxidation of pyragallol, as described by Marklund and Marklund [22]. CAT activity was measured by the published method of Claiborne [23]. The activity of GR enzyme in the erythrocytes was estimated by the method described by Carlberg and Mannervik [24] and GST enzyme was assayed according to Habig [25].

### Determination of pro-inflammatory cytokines level by ELISA

The levels of IL-1β and IL-17 in the serum were determined using an Enzyme Linked Immuno-sorbent Assay employing Human cytokine ELISA set (R&D Systems, USA). ELISA was performed following the manufacturer’s protocol. The sensitivity of the kits ranges from 3.91 to 200pg/ml for IL-1β and 31.20 to 2000pg/ml for IL-17. Briefly, 96-well ELISA plate was coated overnight with either anti-human IL-1β, or IL-17 capture antibodies at 4°C. After washing, wells were blocked with 200 μl of assay diluents (BD Biosciences) at 37°C for 2hr. The plates were finally incubated with the standards and samples at 37°C for 2hr. Detection antibodies were added after washing and incubated at 37°C for 1hr. Further, after washing the plates, streptavidin-HRP conjugate was added to each well and incubated for 1hr at 37°C. Finally, plates were washed and coloured complex was developed using 3,3’,5,5’-tetramethylbenzidine substrate and reaction was stopped after 15min using 2N sulphuric acid. The absorbance was read at 450nm with a microtiter plate reader (Genetix GMB-580). Cytokine levels were determined with the help of standard curve and concentration was expressed as pg/mL.

### Determination of IL-23, IFN-γ and TNF-α expression employing Reverse Transcription-Polymerase Chain Reaction (qRT-PCR)

Total RNA was extracted from the patient’s peripheral blood lymphocytes using PureLink@ RNA mini kit, Invitrogen. Isolated RNA was reverse-transcribed into cDNA by Thermal Cycler (BioRad) using High Capacity cDNA Reverse Transcription Kit, Applied Biosystems, USA. Amplification of the cDNA was performed using an Applied Biosystems 7500 real-time PCR system. Sequences of the forward and reverse primer were as follows: IFN-γ: TCGGTAACTGACTTGAATGTCCA (forward) and TCCTTTTTCGCTTCCCTGTTTT (reverse), IL-23: GACCCACAAGGACTCAAGGAC (forward) and ATGGGGCTATCAGGGAGTAGAG (reverse) and for TNF-α: GCGTTCAGGACACAGACTTG (forward) and TTCATCATTCCAGTTCCGAGTATC synthesized from Eurofins Genomics, India Pvt. ltd. β-actin was used as an endogenous control, for which the primers were: GCCAACCGCGAGAAGATGA (forward primer) and CATCACGATGCCAGTGGTA (reverse primer). RT conditions were: 10min @ 25°C, 120min @ 37°C and 5sec @ 85°C while PCR conditions were: 2min @ 50°C, 10 min at 95°C, and 40 cycles at 15sec @ 95°C followed by 1min @ 60°C.

### Statistical analysis

All the results were expressed as mean ± SD. The statistical significance among the various groups was calculated by unpaired student’s t-test and differences with a p value ≤0.05 were considered to be statistically significant.

## Results

The levels of various clinical and biochemical parameters in T1D patients, grouped on the basis of age, have been summarized in **Table 1** while those of patients grouped on the basis of blood glucose level have been represented in **Table 2**. It can be noted that the mean fasting blood glucose level was found to be elevated in T1D patients as compared to the healthy human subjects in the higher age group. The detailed description of the clinical data of healthy as well as diabetic patients is provided in **S1 File**.

**Table 1:** Biochemical and clinical parameters in healthy and T1D patients of the two age groups ≤15 years and >15 years.

Variable	Healthy Control ≤15 years	T1D ≤15 years	Healthy Control > 15 years	T1D >15 years
**No of individuals (n)**	11	12	14	17
**Age (years)**	11.91 ± 2.43	11.42 ± 2.54	19.43 ± 3.2	19.65 ± 2.55
**BMI (Kg/m**^**2**^**)**	16.2 ± 2.64	12.51 ± 2.46**	20.82 ± 3.02	17.35 ± 3.72^###^
**Fasting blood sugar (mg/dl)**	99.55 ± 14.99	159.92 ± 33.58***	104.36 ± 19.2	178.18 ± 56.27^###^
**HbA1c (%)**	5.04 ± 0.65	9.17 ± 1.33***	4.94 ± 0.69	9.21 ± 1.51^###^
**Serum Creatinine (mg %)**	0.83 ± 0.22	0.74 ± 0.28	0.94 ± 0.25	1.13 ± 0.72
**BUN (mg %)**	12.45 ± 3.91	30.75 ± 19.34	15.57 ± 7.84	37.53 ± 26.18
**AST (IU/L)**	15.45 ± 7.66	9.75 ± 1.42	13.64 ± 4.09	15.12 ± 10.4
**ALT(IU/L)**	14.55 ± 5.76	11.92 ± 2.99	16.86 ± 7.56	17.12 ± 11.2

**p<0.01 and

***p<0.001 vs Healthy Control ≤15 years

^###^p<0.001 vs Healthy Control >15 years

Statistical significance was calculated using unpaired student’s t-test. p≤0.05 is considered statistically significant. Data represented as mean ± Standard Deviation.

**Table 2:** Biochemical and clinical parameters in healthy and T1D patients with fasting blood sugar (BS) ≤160 mg/dl and (BS) >160mg/dl.

Variable	Healthy Control	BS ≤160 mg/dl	BS >160 mg/dl
**No of individuals (n)**	25	14	15
**Age (years)**	16.12 ± 4.75	15 ± 4.51	17.4 ± 4.97
**BMI (Kg/m**^**2**^**)**	18.79 ± 3.65	14.23 ± 4.08**	16.39 ± 3.8
**Fasting blood sugar (mg/dl)**	102.24 ± 17.29	132.36 ± 26.82***	206.33 ± 34.26***
**HbA1c (%)**	4.98 ± 0.66	9.12 ± 1.46***	9.25 ± 1.43***
**Serum Creatinine (mg%)**	0.89 ± 0.24	0.81 ± 0.23	0.98 ± 0.58
**BUN (mg%)**	14.2 ± 6.49	28.86 ± 17.71	38.07 ± 28.58
**AST (IU/L)**	14.44 ± 5.86	12.71 ± 5.66	13.07 ± 10.48
**ALT(IU/L)**	15.84 ± 6.79	14.79 ± 5.06	15.27 ± 11.76

**p<0.01 and

***p<0.001 vs Healthy Control

Statistical significance was calculated using unpaired student’s t-test. p≤0.05 is considered statistically significant. Data represented as mean ± Standard Deviation.

**Table 3** and **Table 4** summarize the levels of various oxidative stress markers, FRAP values, total erythrocyte thiols and non-enzymatic antioxidants in both healthy as well as T1D patients grouped on the basis of age and blood sugar level respectively. FRAP values were significantly lower in both T1D age groups as compared to respective healthy controls (p<0.01and p<0.001). A generalized decrease in the FRAP values was found with increasing age and blood sugar level (p<0.01 and p<0.05 respectively). Malondialdehyde levels were found significantly elevated in T1D patients belonging to both age groups as compared to the age matched healthy controls. The MDA levels were found to augment significantly with age (p<0.01). Protein carbonyl content in the two diabetic age groups was also significantly higher as compared to the age matched healthy control groups, however, no significant difference was observed among the two hyperglycaemic groups (p>0.05). Similar to the decline in protein carbonyl content, Vitamin C level was also lower in the patients grouped on the basis of age with higher significance in the >15 years age group compared to the respective control group.

**Table 3:** Biochemical parameters in Healthy and T1D patients of the two age groups ≤15 years and >15 years.

Biochemical Parameters	Healthy Control (≤15 years)	T1D (≤15 years)	Healthy Control (>15 years)	T1D (>15 years)
**FRAP (μmol/L)**	817.35 ± 165.28	635.51 ±129.36**	792.86 ± 132.69	518.84 ±102.25^###^
**MDA (nmol/mL)**	1.78 ± 0.67	2.53 ± 0.45 **	2.01 ± 0.71	3.11 ± 0.53^###^
**Protein Carbonyl content (nmol/mg protein)**	2.07 ± 0.48	3 ± 0.56 ***	2.1 ± 0.53	3.59 ± 0.6^###^
**Vitamin C (ng/mL)**	20.79 ± 5.74	13.23 ± 4.72**	19.59 ± 5.93	9.8 ± 3.24^###^
**Reduced Glutathione (nmol/mL)**	186.87 ± 41.46	136.42 ± 23.52**	173.2 ± 44.1	112.85 ± 3.90^###^
**Total SH groups (nmol/mg protein)/**	8.83 ± 2.9	6.67 ± 1.78*	8.26 ± 2.02	4.93 ± 0.99^###^

*p≤0.05

**p<0.01 and

***p<0.001 vs Healthy Control ≤15 years

^###^p<0.001 vs Healthy Control >15 years

Statistical significance was calculated using unpaired student’s t-test. p≤0.05 is considered statistically significant. Data represented as mean ± Standard Deviation.

**Table 4:** Biochemical parameters of healthy control subjects and T1D patients with fasting blood sugar (BS) ≤160 mg/dl and >160mg/dl.

Biochemical Parameters	Healthy Control	Blood Sugar (≤160 mg/dl)	Blood Sugar (>160 mg/dl)
**FRAP (μmol/L)**	803.63 ± 145.17	610.32 ± 101.25***	513.47 ± 136.48***
**MDA (nmol/mL)**	1.91 ± 0.69	2.69 ± 0.84**	3.37 ± 0.89***
**Protein Carbonyl content (nmol/mg protein)**	2.09 ± 0.50	3.25 ± 0.70***	3.43 ±0.60***
**Vitamin C (ng/mL)**	20.12 ± 5.80	11.30 ± 3.90***	11.14 ± 4.62***
**Reduced Glutathione (nmol/mL)**	179.22 ± 42.63	132.57 ± 23.28***	113.30 ± 15.47***
**Total SH groups (nmol/mg protein)/**	8.18 ± 2.41	6.26 ± 1.86**	5.08 ± 1.09***

**p<0.01 and

***p<0.001 vs Healthy Control

Statistical significance was calculated using unpaired student’s t-test. p≤0.05 is considered statistically significant. Data represented as mean ± Standard Deviation.

Furthermore, the level of non-enzymatic antioxidant GSH was found to be significantly reduced in T1D patients of both age groups as compared to healthy controls (p<0.01 and p<0.001). A notable decrease was found in the higher age groups. With increased blood sugar level, GSH level further lowered in statistically significant manner (p<0.001). Erythrocyte total thiols in the diabetic age groups showed a significant decline as compared to the age matched healthy control groups. Similar to the pattern seen in FRAP values, total thiols showed greater decrease in >15 years age group patients (p<0.01). Although less significant, but decline was observed in patients with high blood glucose (p<0.05) as well.

**Fig 1:**
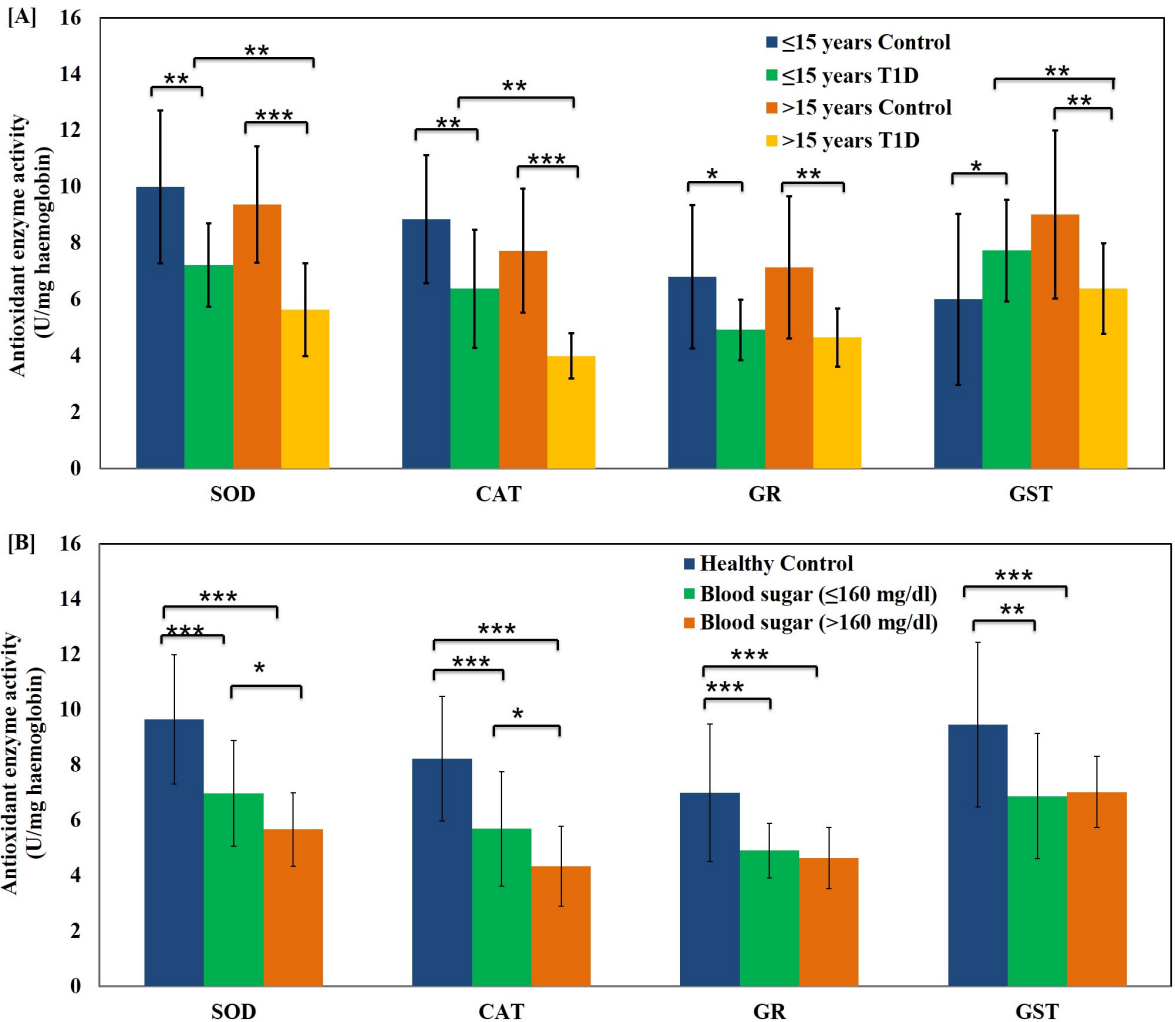
Shift in the activity of erythrocyte antioxidant enzymes with age and glycemic condition of the T1D patients. (A) Activity of erythrocyte antioxidant enzymes SOD, CAT, GR and GST in healthy control subjects and T1D patients of age groups ≤15 years and >15 years. (B) Activity of erythrocyte antioxidant enzymes SOD, CAT, GR and GST in healthy control subjects and T1D patients with fasting blood sugar (BS) ≤160 mg/dl and >160 mg/dl. Data represent mean ± S.D. Statistical significance was determined by unpaired student’s t-test and represented as *p≤0.05, **p<0.01 and ***p<0.001. p≤0.05 was considered statistically significant.

**Fig 2:**
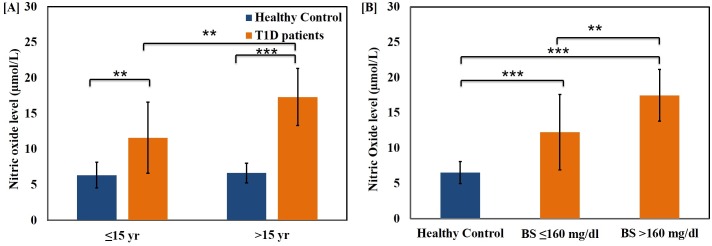
Variations in the level of plasma Nitric oxide with age and glycemic condition of the T1D patients. Plasma Nitric Oxide levels in healthy control subjects and T1D patients grouped on the basis of (A) age (≤15 years and >15 years) and (B) fasting blood glucose levels (≤160 mg/dl and >160 mg/dl). Data represent mean ± S.D. Statistical significance was determined by unpaired student’s t-test and represented as *p≤0.05, **p<0.01 and ***p<0.001. p≤0.05 was considered statistically significant.

The activity of erythrocyte antioxidant enzymes SOD, CAT, GR and GST was found to be significantly suppressed in the diabetic patients as compared to age matched healthy volunteers. Interestingly, there was significant decrease in the SOD, CAT and GST activity in diabetic subjects of both age groups (p<0.01) **(Fig 1[A]).** The activity of all the antioxidant enzymes was significantly diminished in the two hyperglycaemic groups. Although SOD and CAT showed relatively less significant decline in the high blood glucose group (p<0.05), but GR and GST activity did not change (p>0.05) **(Fig 1[B]).** Nitric oxide levels were significantly higher in both the diabetic age groups as compared to the corresponding healthy control groups **(Fig 2[A])**. Like other oxidative stress parameters, NO level also augmented with age (p<0.05), but the difference was more significant in higher age group T1D patients (p<0.001). Patients with blood sugar >160 mg/dl showed significantly higher NO levels as compared to those with blood sugar ≤160 mg/dl (p<0.01) **(Fig 2[B]).**

**Fig 3:**
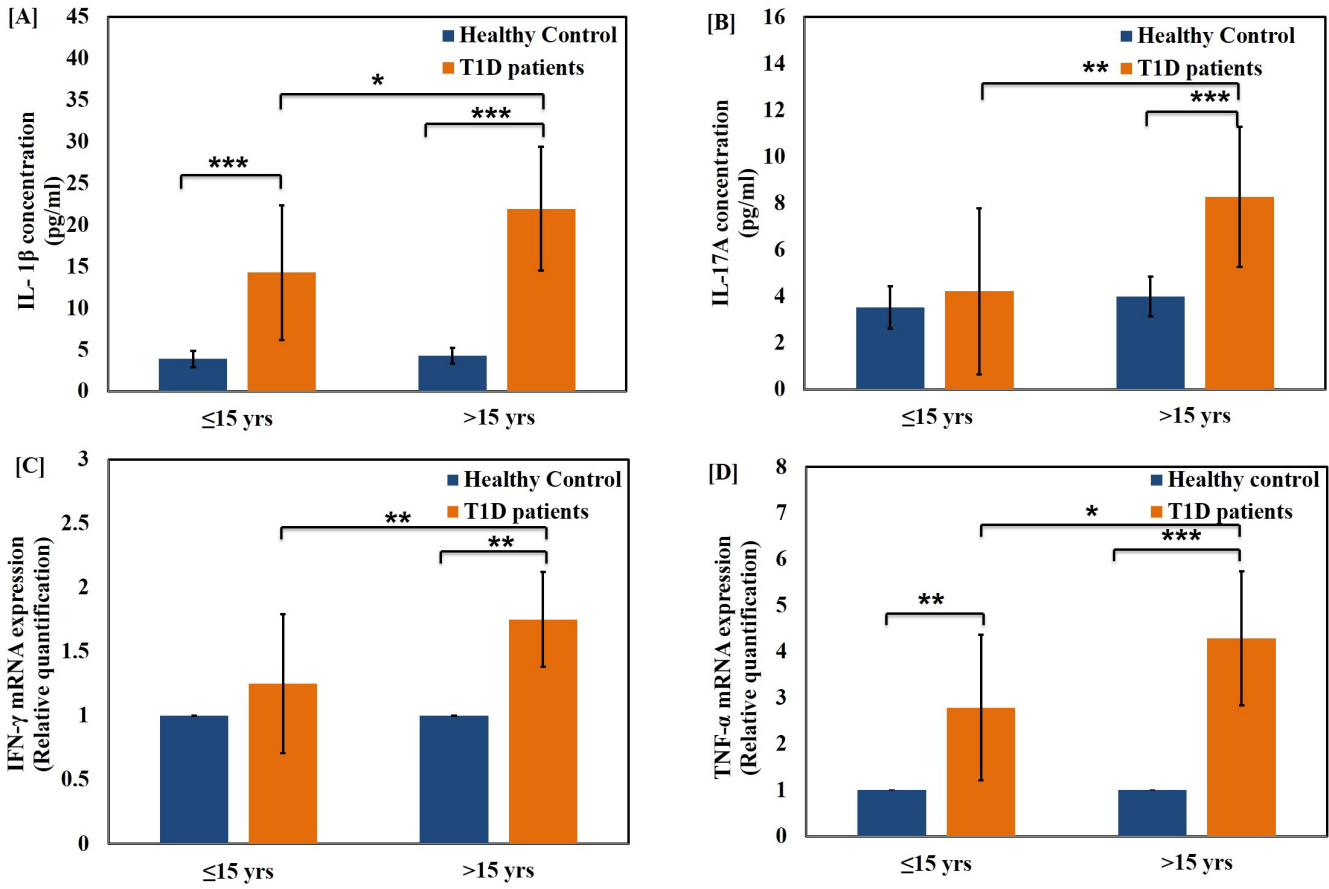
Changes in the level of pro-inflammatory cytokines in the T1D patients with age. Serum levels of pro-inflammatory cytokines (A) IL-1β (B) IL-17A and expression fold in the whole blood of pro-inflammatory cytokines (C) IFN-γ and (D) TNF-α in healthy control subjects and T1D patients of age groups ≤15 years and >15 years. Data represent mean ± Standard Deviations from three separate experiments. Statistical significance was determined by unpaired student’s t-test and represented as *p≤0.05, **p<0.01 and ***p<0.001. p≤0.05 was considered statistically significant.

**Fig 4:**
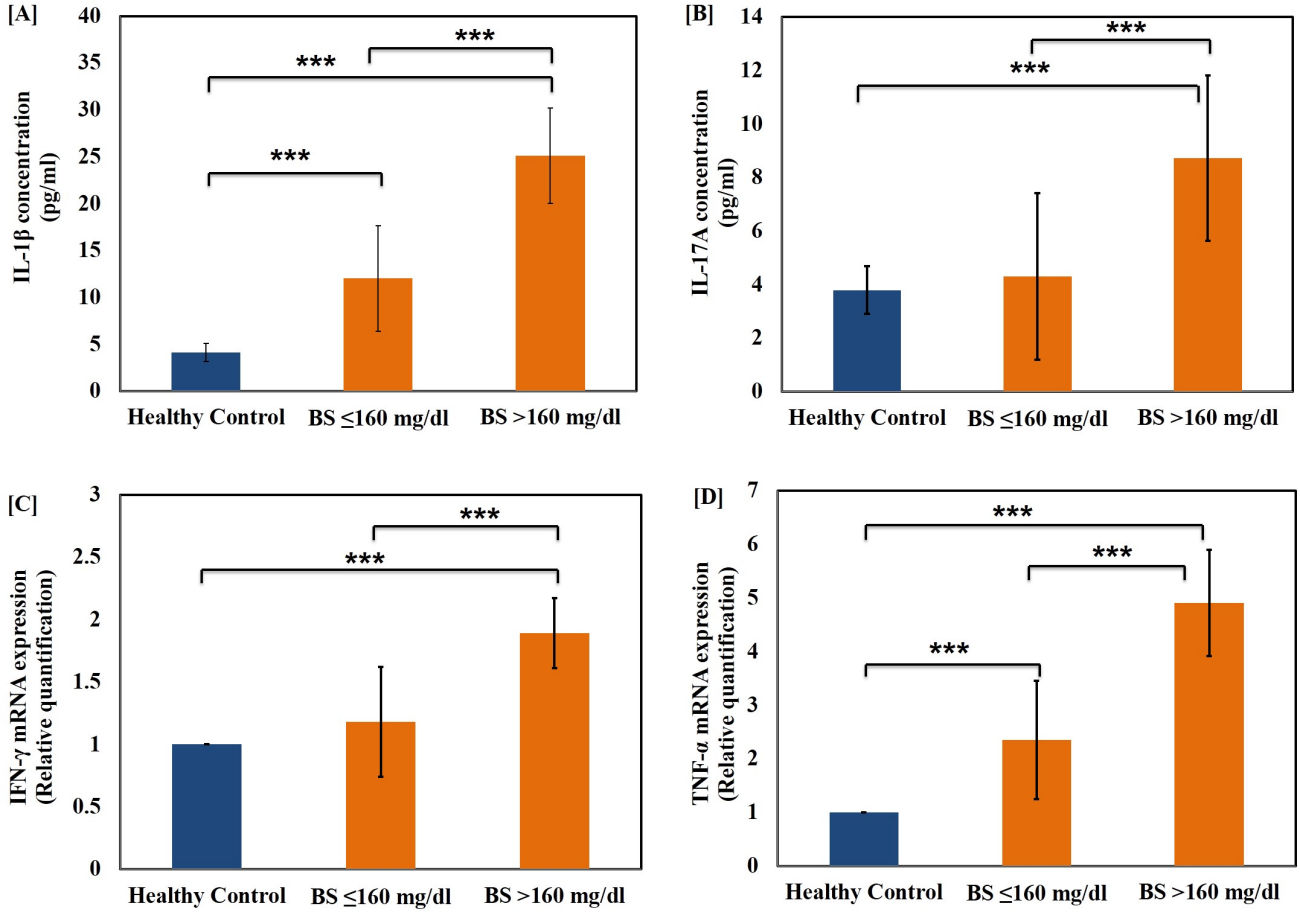
Changes in the level of pro-inflammatory cytokines in the T1D patients with varying blood glucose threshold. Serum levels of pro-inflammatory cytokines (A) IL-1β (B) IL-17A and expression level of cytokines (C) IFN-γ and (D) TNF-α in healthy control subjects and T1D patients with fasting blood sugar (BS) ≤160 mg/dl and >160 mg/dl. Data represent mean ± Standard Deviation from three separate experiments. Statistical significance was determined by unpaired student’s t-test and represented as *p≤0.05, **p<0.01 and ***p<0.001. p≤0.05 was considered statistically significant.

**Fig 5:**
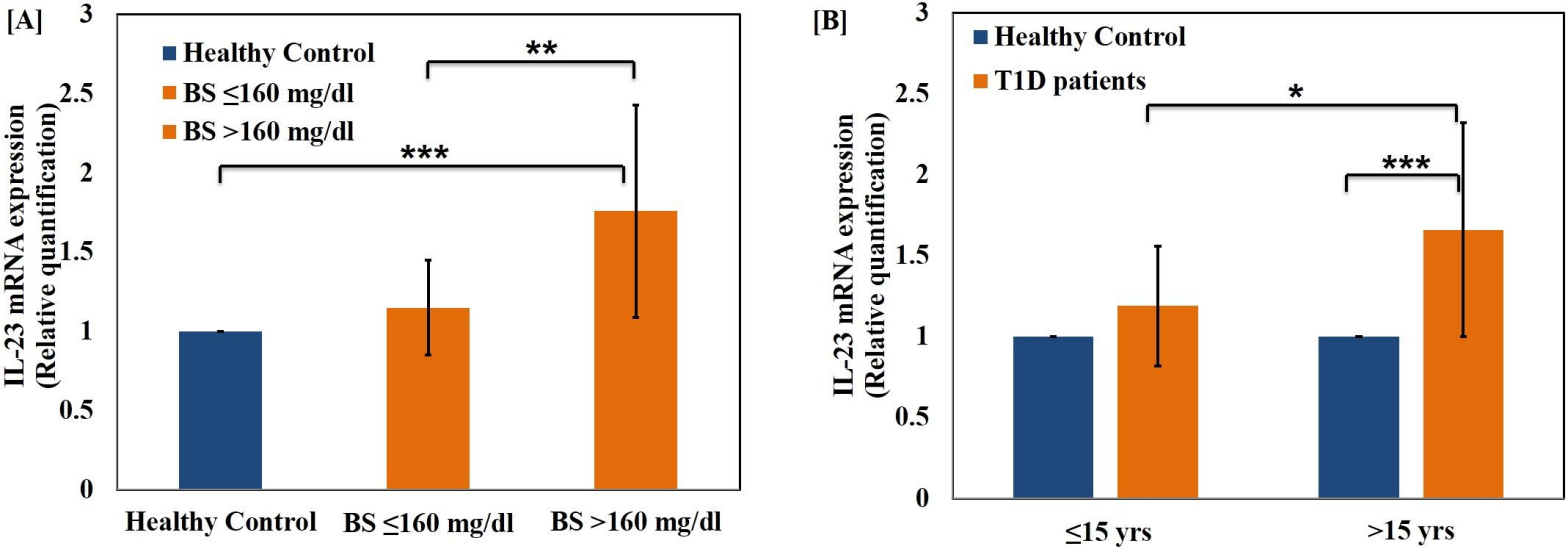
Up-regulation of IL-23 expression in T1D patients. mRNA expression level of cytokine IL-23 in T1D patients, (A) with varying blood sugar level (B) with the progression of age, when compared to healthy control subjects. Data represent mean ± Standard Deviation from three separate experiments. Statistical significance was determined by unpaired student’s t-test. The p values represented as *p≤0.05, **p<0.01 and ***p<0.001 were considered statistically significant.

The level of pro-inflammatory cytokines, IL-1β **(Fig 3[A])** and TNF-α **(Fig 3[D])** were also determined in T1D patients belonging to both age groups. The cytokines were up-regulated significantly in both age groups as compared to age-matched healthy control groups. The TNF-alpha mRNA expression was significantly elevated in both higher age group (p<0.001) as well as lower age group (p<0.01) when compared to respective healthy control groups. Furthermore, the level of these cytokines were significantly elevated in both the hyperglycaemic groups as compared to healthy controls (p<0.001). The difference between the two groups was found to be highly significant (p<0.001) as shown in **Fig 4[A]** and **Fig 4[D].** We further evaluated the serum level of IL-17A, a Th17 specific pro-inflammatory cytokine and mRNA expression of the cytokines IFN-γ in the T1D patients as represented in **Fig 3B and 3C** and **Fig 4B and 4C** respectively. The levels of cytokine IL-17 were found to be significantly higher in the age group >15 years and hyper-glycaemic group >160 mg/dl (p<0.001) as compared to healthy controls. The difference in the cytokine expression among the two hyper-glycaemic groups was of greater significance for various cytokines tested (p<0.001). Among the two groups, the patients with blood sugar level BS≥160 mg/dl were found to possess significantly higher IL-23 level when compared to the group with BS≤160 mg/dl as well as healthy control subjects of matching age groups as represented in **Fig 5[A]** while there was low significant change with age as shown in **Fig 5[B]**. It can be inferred that the level of both IL-17 and IL-23 were up-regulated significantly and had great deal of correlation with T1D pathogenesis. The interplay of these inflammatory cytokines facilitates insulin secretory dysfunction and may promote damage of insulin producing β-cells, resulting in the onset of T1D.

## Discussion

In general, destruction of insulin producing β cells in T1D patients ensues in elevated plasma sugar level. The chronic hyper-glycaemia causes chemical alterations in proteins, lipids and DNA on one hand and also facilitate generation of reactive oxygen species (ROS) mediated enhanced oxidative stress on the other. This is generally accompanied by impaired antioxidant defence in T1D patients [26,27,9]. Keeping the above fact into consideration, the present study aimed to evaluate the biochemical markers of oxidative stress and inflammatory cytokines in T1D patients in Aligarh region of North India. The special emphasis was given to the patients with poorly controlled diabetes and had average HbA1c values close to ~9. We stratified the patients according to age above and below 15 years since the average age at which children attain puberty is around 15–16 years. This is a very crucial stage of life since the individual experiences a great deal of hormonal changes and also exposed to pronounced oxidative stress. The induction of oxidative stress during puberty in type 1 diabetic patients has been reported in a study by Elhadd *et al* [28]. After puberty (>15 years of age), the accompanying hormonal development makes the glycemic control more obscure, and probably, this is the time when managing type 1 diabetes becomes the most challenging. Apart from age based categorization, patients were also stratified on the basis of the level of fasting blood sugar level above and below 160 mg/dl. The higher level of fasting blood glucose can directly promote oxidative stress in patients, thus 160 mg/dl was chosen as a critical fasting blood glucose level when the patients no longer fall into the category of well controlled diabetes. Our strategy here was to assess the oxidative stress and inflammation in the patients whose fasting blood glucose level remained within this critical value as compared to those whose fasting blood sugar crossed this threshold. According to a study conducted by Menon V et al, the balance between oxidative and antioxidant processes appears to be sensitive to plasma glucose level [29].

Malondialdehyde (MDA) can be regarded as an important and reliable biomarker of oxidative stress [30,31,32]. MDA is the end product of polyunsaturated fatty acid peroxidation. The plasma membrane lipids become more susceptible to oxidation with age, presumably due to the effect of hyperglycaemia as well as aging mediated generation of free radicals in older diabetic patients. Besides, the elevated plasma glucose concentration leads to enhanced lipid peroxide formation, accounting for higher MDA levels in patients with blood sugar >160 mg/dl [33]. The enhanced Protein Carbonyl (PC) formation in T1D as compared to healthy control subjects is indicative of increased free radical mediated damage to proteins in diabetics and oxidized proteins are constituents of lesser active enzymes involved in metabolism [34, 35]. The increase in PC content of patients with age is a direct consequence of enhanced oxidative stress as the antioxidant capacity of the body fails in older people [36, 37].

The total antioxidant capacity of plasma as determined by the FRAP values were found to be significantly decreased in T1D patients which indicates an impaired defense mechanism against oxidative stress [33,38,39]. Thiols when present in adequate amounts in the erythrocyte membrane facilitate proper protein folding and stability. The oxidative damage to the thiol groups destabilizes the erythrocyte membrane proteins [40]. A significant decline in erythrocyte total SH groups with age can be attributed to the ROS mediated oxidative damage to membrane proteins may also result in loss of membrane fluidity [41].

We observed a significantly lower level of plasma GSH in T1D patients compared to healthy controls. It can be explained on the premise that due to impaired transporter function intracellular glutathione concentration decreased in plasma and erythrocytes of diabetic patients in age dependent manner [9,40,42,43]. GSH depletion in the plasma might also occur due to unaltered fractional glutathione synthesis rates depending upon good or poor glycemic control, increased glutathione utilization and enhanced oxidation of Glutathione (GSH) to Glutathione disulphide (GSSG) [9]. This reduction was more apparent in high blood glucose group which significant negative correlation between fasting plasma glucose and plasma GSH values [43]. The level of vitamin C, another essential non-enzymatic antioxidant, was found to be significantly lowered in the plasma of T1D patients compared to age matched healthy controls [44]. Decreased vitamin C levels may be due to excessive excretion, reduced intake in the diet or increased oxidation as a consequence of enhanced free radical activity. There is no significant change in Vitamin C levels in the high blood glucose group which is in contrast with a study showing significant negative correlation between plasma vitamin C levels and blood glucose [26].

In a line similar to other reports, the data of the present study suggested a significant elevation in the level of basal nitric oxide (NO) in the plasma of T1D patients [32,45,46]. Under hyperglycaemia-induced oxidative stress, NO is synthesized in bulk by the activation of iNOS in the β-cells, subjecting the pancreatic cells to systemic nitrosative stress. NO causes injury to the β-cells, thereby enhancing inflammation and apoptosis [45]. The decreased total antioxidant capacity ensuing in enhanced oxidative stress may cause further increase in level of plasma NO in T1D children of older age. We found increased NO levels in patients with blood glucose >160 mg/dl which is consistent with another study suggesting that elevated concentrations of glucose can lead to up-regulation of NOS gene product that eventually causes NO release in human aortic endothelial cells [47].

We assessed expression of antioxidant enzymes SOD, CAT, GR and GST in the erythrocyte of T1D patients. The onset of diabetes causes diminished expression of these enzymes in T1D patients as compared to their age-matched healthy controls. The diminutive activity of antioxidant enzymes SOD and CAT in T1D patients is indicative of free radical mediated injury. Erythrocytes are continuously flooded with hydrogen peroxide and superoxide radicals are dismutated into hydrogen peroxide by SOD enzyme [26]. The superoxide radicals disabled enzymes failed to scavange free radicals. Some reports suggest that hyperglycaemia lead to SOD glycation, thereby rendering it lesser active than the non-glycated form. SOD activity in older T1D individuals was found to be significantly less than their younger counterparts although Ruiz C *et al* found no significant effect of age on the activity of SOD [48,49].

There was substantial decrease in erythrocyte CAT activity in T1D patients as compared to healthy controls [28]. Elevated glucose levels as well as increased hydrogen peroxide may induce in activation of catalase [50]. The decline in GR activity is supported by several studies [51], according to which GR may be highly susceptible to structural damage *in vivo* by ROS and marked for proteolytic degradation. Several earlier studies have reported a negative correlation of GR and GST activity with age [52], however, we could not find any significant decline in GR activity in the higher age groups. This could be attributed to the fact that the median age in the two groups lie in a relatively younger age group (comprising of 11.43±2.54 for <15 years and 19.65±2.55 for >15 years age group). In a study conducted on STZ-induced type 1 diabetic rats, GST activity was found to decrease. GST mRNA is diminished due to oxidation of various transcription factors essential for antioxidant enzyme transcription or may be due to destabilization of mRNA owing to its decreased half-lives because of increased oxidative stress [53].

Hyperglycemia is the specific metabolic feature of diabetes and is considered responsible for wide spread cellular and molecular damage [54]. Cytokines are primary mediators of inflammation, which act by controlling innate, adaptive immune responses as well as tissue damage, defense, repair, and remodeling [55]. Cells, which poorly regulate intracellular glucose, are particularly vulnerable to hyperglycemia [56]. The intracellular redox status is closely linked to the levels of pro-inflammatory cytokines, IL-1β, IL-23 and TNF-α which are the main components of inflammatory responses [57]. IFN-γ has also been shown to be associated with inflammation while TNF-α has been studied extensively for its role in the inflammatory process and production of ROS [58]. Keeping the above facts into consideration, we evaluated the level of IL-1β and IL-17 using ELISA while IFN-γ, IL-23 and TNF-α mRNA expression fold change was determined using qRT-PCR. We found that IL-1β (pro-inflammatory cytokine) levels were significantly higher in both diabetic groups as compared to the healthy control subjects. The IL-1β is generally expressed by in-filtering macrophages, once activated they synthesize higher amount of nitric oxide as well. Interestingly, there were remarkable differences in both NO and cytokine levels in the patients of higher age groups with glucose levels higher than 160 mg/dl when compared with lower age groups with glucose level upto 160 mg/dl.

IFN-γ induced islet beta-cell destruction by accelerating activation-induced cell death (apoptosis) [59]. Further, by up-regulating the expression of adhesion molecules, interferon-gamma facilitates homing of auto reactive leukocytes in the pancreas that causes further beta-cell destruction. Besides, IFN-γ production by lymphocytes is dependent upon secretion of IL-1 by accessory adherent cells [60]. Our data revealed that pro-inflammatory markers viz. IL-1β, TNF-α, IL-23 and IFN-γ has a strong correlation with the age and level of blood glucose. Nevertheless, there was significant up-regulation in the patients of higher age groups and glucose levels higher than 160 mg/dl when compared with lower age groups and glucose level upto 160 mg/dl as well as with healthy control subjects. Interestingly, our data indicated that IFN- γ levels were comparable in the lower age group and glucose level upto 160 mg/dl in comparison with the healthy control subjects. The level of IFN-γ is significantly up-regulated in the patients of higher age groups with glucose levels higher than 160 mg/dl as compared with lower age groups and glucose level upto 160 mg/dl as well as with the healthy control subjects.

Numerous studies on T1DM have already been published which emphasize on the status of oxidative stress and inflammation in patients with good and poor glycemic control in terms of low and high HbA1c values. In contrast, our study focussed on patients with poor glycemic control that were divided into two groups, one with moderate blood glucose levels and the other with high blood glucose levels. We have tried to elucidate the differences in stress and glycemic condition between the two groups of patients (with elevated HbA1c), when their fasting blood glucose level was higher or lower from a specific value.

IL-1β has been reported to be involved in augmenting the expression of inducible nitric oxide synthase (iNOS), as a result of which free radical nitric oxide generation takes place [61]. The present state of knowledge proposes IL-17 as a new participant in inflammation process [62]. Our results suggest that serum IL-17A levels follow the pattern similar to that of the IFN-γ level in the both T1D groups. To the best of our knowledge, this is one of the few studies that elucidate the plausible role of interleukins viz. IL-17, IL-23 and IFN-γ in the pathogenesis of T1D and assessed its correlation with age and glycemic condition of patients.

In general, IL-23 is produced by inflammatory myeloid cells and influences the development of IL-17 producing T helper (TH17) cell responses [63]. We observed an accordant pattern of levels of both the cytokines in T1D patients. The present study highlights pro-inflammatory cytokine interplay which leads to the development of diabetes. Our data on IL-23 suggest that during T1D disease progression pro-inflammatory cytokines play a crucial role and most likely initiate and propagate inflammatory cascades which damages the beta cells of the pancreas with the recruitment CD8^+^ T-cells [64]. In concordance with the earlier published reports regarding role of the two cytokines in various auto-immune diseases, we observed synergistic interplay between IL-23, IL-17 and TNF-α [65].

Alteration in the level of pro-inflammatory cytokine (especially the IL-17A, IL-23 and IFN-γ) in T1D patients imply its significance as a potential pathogenesis marker, thereby extending the threat of developing diabetes related complications later in life. This knowledge can be render useful in better prediction of the disease progression after diagnosis with T1D.

## Conclusion

The data of the present study suggest a strong association between advancement of T1D with elevated level of pro-inflammatory cytokines as well as oxidative stress markers. It was interesting to notice that although HbA1c was high in most of the patients, yet there was a pronounced effect of fasting plasma glucose levels on the studied parameters, i.e., high HbA1c when accompanied with low fasting plasma glucose could keep inflammation and oxidative stress under control to a considerable extent. The observed elevation in various biochemical parameters specially elevated NO levels in T1D patients can be attributed to the activation of macrophages which in turn leads to enhanced ROS generation. To the best of our knowledge, the present work for the first time shows correlation between pro-inflammatory cytokines and oxidative stress markers with age and blood glucose level in T1D human subjects having poor glycemic control with average HbA1c values above 9. The cytokines viz. IL-23, IFN-γ and IL-17A are believed to contribute to the pathogenesis of T1D on the basis of age and glycemic condition of the patients. Further, prospective in-depth studies are needed to validate this threshold and may provide new insights into the immunological events which occur during T1D.

## Supporting Information

S1 File Contains clinical data of both healthy human subjects as well as data pertaining to various diabetic patients groups in the form of Tables. **Table A** contains clinical data of all the healthy subjects taken as controls, **Table B** contains clinical data of healthy control subjects in the age group ≤15 years, **Table C** contains clinical data of healthy control subjects in the age group >15 years, **Table D** contains clinical data of T1D patients in the age group ≤15 years, **Table E** contains **c**linical data of T1D patients in the age group >15 years, **Table F** contains clinical data of T1D patients with fasting blood sugar ≤160 mg/dl and **Table G** contains clinical data of T1D patients with fasting blood sugar >160 mg/dl.
(DOCX)
