# Supplementary material for: Role of Pro-Inflammatory Cytokines and Biochemical Markers in the Pathogenesis of Type 1 Diabetes: Correlation with Age and Glycemic Condition in Diabetic Human Subjects
Source: PLoS One. 2016 Aug 30;11(8):e0161548. doi: 10.1371/journal.pone.0161548 (PMC5004869; doi:10.1371/journal.pone.0161548)
Supplement: S1 File — Table A contains clinical data of all the healthy subjects taken as controls, Table B contains clinical data of healthy control subjects in the age group ≤15 years, Table C contains clinical data of healthy control subjects in the age group >15 years, Table D contains clinical data of T1D patients in the age group ≤15 years, Table E contains clinical data of T1D patients in the age group >15 years, Table F contains clinical data of T1D patients with fasting blood sugar ≤160 mg/dl and Table G contains clinical data of T1D patients with fasting blood sugar >160 mg/dl. (DOCX) [file pone.0161548.s001.docx]

**S1 Table A:** Clinical data of all the healthy subjects taken as controls.

| **Control No.** | **Gender** | **Age (years)** | **Fasting**  **Blood**  **Sugar**  **(mg/dl)** | **HbA1c (%)** | **BMI (Kg/m^2^)** | **Serum Creatinine**  **(mg%)** | **BUN (mg%)** | **AST (IU/L)** | **ALT (IU/L)** |
| --- | --- | --- | --- | --- | --- | --- | --- | --- | --- |
| 1 | M | 11 | 107 | 4.8 | 13.53 | 0.7 | 18 | 8 | 18 |
| 2 | M | 9 | 95 | 4.2 | 12.82 | 0.6 | 14 | 12 | 11 |
| 3 | F | 11 | 118 | 4.9 | 14.26 | 0.45 | 10 | 17 | 10 |
| 4 | M | 13 | 85 | 4 | 18.8 | 1 | 9 | 10 | 8 |
| 5 | F | 15 | 98 | 5.4 | 17.93 | 0.85 | 12 | 9 | 9 |
| 6 | F | 14 | 103 | 5.1 | 19.27 | 0.74 | 9 | 29 | 22 |
| 7 | F | 8 | 112 | 4.6 | 12.53 | 0.9 | 11 | 21 | 19 |
| 8 | F | 10 | 106 | 4.9 | 15.27 | 0.7 | 19 | 10 | 25 |
| 9 | M | 11 | 118 | 5.6 | 17.38 | 1.2 | 9 | 17 | 10 |
| 10 | M | 14 | 78 | 5.8 | 16.82 | 1.1 | 9 | 28 | 16 |
| 11 | F | 15 | 75 | 6.1 | 19.64 | 0.9 | 17 | 9 | 12 |
| 12 | M | 22 | 111 | 4.1 | 22.15 | 0.67 | 16 | 8 | 21 |
| 13 | F | 16 | 89 | 4.2 | 18.47 | 0.8 | 10 | 11 | 17 |
| 14 | F | 19 | 96 | 5.5 | 23.68 | 0.75 | 22 | 17 | 10 |
| 15 | M | 20 | 88 | 5.6 | 19.92 | 1.3 | 11 | 21 | 13 |
| 16 | M | 17 | 107 | 4.9 | 18.38 | 0.9 | 14 | 18 | 35 |
| 17 | F | 16 | 79 | 5.8 | 20.63 | 0.57 | 10 | 16 | 12 |
| 18 | M | 25 | 94 | 6.2 | 24.9 | 0.8 | 8 | 10 | 19 |
| 19 | M | 26 | 88 | 4.3 | 25.95 | 0.96 | 21 | 12 | 8 |
| 20 | M | 19 | 119 | 4.8 | 17.37 | 0.84 | 16 | 17 | 16 |
| 21 | M | 16 | 114 | 5 | 15.85 | 0.9 | 38 | 9 | 14 |
| 22 | M | 17 | 155 | 5.1 | 23.14 | 1.3 | 11 | 18 | 24 |
| 23 | F | 21 | 99 | 5.4 | 22.59 | 1.4 | 19 | 11 | 26 |
| 24 | F | 20 | 120 | 4.3 | 17.92 | 0.9 | 13 | 13 | 12 |
| 25 | M | 18 | 102 | 4 | 20.47 | 1.1 | 9 | 10 | 9 |

**S1 Table B:** Clinical data of healthy control subjects in the age group ≤15 years.

| **Control**  **No.** | **Gender** | **Age (years)** | **Fasting**  **Blood**  **Sugar**  **(mg/dl)** | **HbA1c (%)** | **BMI (Kg/m^2^)** | **Serum Creatinine**  **(mg%)** | **BUN (mg%)** | **AST (IU/L)** | **ALT (IU/L)** |
| --- | --- | --- | --- | --- | --- | --- | --- | --- | --- |
| 1 | M | 11 | 107 | 4.8 | 13.53 | 0.7 | 18 | 8 | 18 |
| 2 | M | 9 | 95 | 4.2 | 12.82 | 0.6 | 14 | 12 | 11 |
| 3 | F | 11 | 118 | 4.9 | 14.26 | 0.45 | 10 | 17 | 10 |
| 4 | M | 13 | 85 | 4 | 18.8 | 1 | 9 | 10 | 8 |
| 5 | F | 15 | 98 | 5.4 | 17.93 | 0.85 | 12 | 9 | 9 |
| 6 | F | 14 | 103 | 5.1 | 19.27 | 0.74 | 9 | 29 | 22 |
| 7 | F | 8 | 112 | 4.6 | 12.53 | 0.9 | 11 | 21 | 19 |
| 8 | F | 10 | 106 | 4.9 | 15.27 | 0.7 | 19 | 10 | 25 |
| 9 | M | 11 | 118 | 5.6 | 17.38 | 1.2 | 9 | 17 | 10 |
| 10 | M | 14 | 78 | 5.8 | 16.82 | 1.1 | 9 | 28 | 16 |
| 11 | F | 15 | 75 | 6.1 | 19.64 | 0.9 | 17 | 9 | 12 |

**S1 Table C:** Clinical data of healthy control subjects in the age group >15 years.

| **Control**  **No.** | **Gender** | **Age (years)** | **Fasting**  **Blood**  **Sugar**  **(mg/dl)** | **HbA1c (%)** | **BMI (Kg/m^2^)** | **Serum Creatinine**  **(mg%)** | **BUN (mg%)** | **AST (IU/L)** | **ALT (IU/L)** |
| --- | --- | --- | --- | --- | --- | --- | --- | --- | --- |
| 1 | M | 22 | 111 | 4.1 | 22.15 | 0.67 | 16 | 8 | 21 |
| 2 | F | 16 | 89 | 4.2 | 18.47 | 0.8 | 10 | 11 | 17 |
| 3 | F | 19 | 96 | 5.5 | 23.68 | 0.75 | 22 | 17 | 10 |
| 4 | M | 20 | 88 | 5.6 | 19.92 | 1.3 | 11 | 21 | 13 |
| 5 | M | 17 | 107 | 4.9 | 18.38 | 0.9 | 14 | 18 | 35 |
| 6 | F | 16 | 79 | 5.8 | 20.63 | 0.57 | 10 | 16 | 12 |
| 7 | M | 25 | 94 | 6.2 | 24.9 | 0.8 | 8 | 10 | 19 |
| 8 | M | 26 | 88 | 4.3 | 25.95 | 0.96 | 21 | 12 | 8 |
| 9 | M | 19 | 119 | 4.8 | 17.37 | 0.84 | 16 | 17 | 16 |
| 10 | M | 16 | 114 | 5 | 15.85 | 0.9 | 38 | 9 | 14 |
| 11 | M | 17 | 155 | 5.1 | 23.14 | 1.3 | 11 | 18 | 24 |
| 12 | F | 21 | 99 | 5.4 | 22.59 | 1.4 | 19 | 11 | 26 |
| 13 | F | 20 | 120 | 4.3 | 17.92 | 0.9 | 13 | 13 | 12 |
| 14 | M | 18 | 102 | 4 | 20.47 | 1.1 | 9 | 10 | 9 |

**S1 Table D:** Clinical data ofT1D patients in the age group ≤15 years.

| **Patient No.** | **Age (years)** | **Gender** | **Fasting**  **Blood Sugar (mg/dl)** | **HbA1c (%)** | **BMI (Kg/m^2^)** | **Serum Creatinine**  **(mg%)** | **BUN**  **(mg%)** | **AST**  **(IU/L)** | **ALT**  **(IU/L)** |
| --- | --- | --- | --- | --- | --- | --- | --- | --- | --- |
| 1 | 15 | F | 186 | 10.8 | 17.3 | 0.7 | 25 | 10 | 13 |
| 2 | 12 | F | 199 | 9.5 | 15.3 | 0.46 | 36 | 12 | 15 |
| 3 | 10 | M | 154 | 7.6 | 11.08 | 0.8 | 34 | 9 | 10 |
| 4 | 12 | F | 145 | 9.3 | 13.59 | 0.7 | 7 | 9 | 10 |
| 5 | 9 | M | 167 | 8.1 | 11.76 | 0.9 | 33 | 10 | 11 |
| 6 | 13 | F | 152 | 10.2 | 11.05 | 0.9 | 8 | 9 | 8 |
| 7 | 8 | F | 189 | 11.9 | 12.16 | 0.37 | 40 | 11 | 17 |
| 8 | 15 | F | 215 | 8.9 | 14.81 | 0.93 | 28 | 7 | 9 |
| 9 | 12 | F | 118 | 7.4 | 8.16 | 0.5 | 9 | 9 | 10 |
| 10 | 13 | M | 154 | 9 | 12.82 | 1.4 | 78 | 12 | 17 |
| 11 | 11 | F | 142 | 8 | 11.7 | 0.67 | 28 | 10 | 11 |
| 12 | 7 | M | 98 | 9.3 | 10.37 | 0.54 | 43 | 9 | 12 |

**S1 Table E:** Clinical data of T1D patients in the age group >15 years.

| **Patient**  **No.** | **Age (years)** | **Gender** | **Fasting**  **Blood**  **Sugar**  **(mg/dl)** | **HbA1c**  **(%)** | **BMI**  **(Kg/m^2^)** | **Serum Creatinine**  **(mg%)** | **BUN**  **(mg%)** | **AST**  **(IU/L)** | **ALT**  **(IU/L)** |
| --- | --- | --- | --- | --- | --- | --- | --- | --- | --- |
| 1 | 16 | M | 215 | 9.1 | 13.67 | 0.9 | 34 | 17 | 21 |
| 2 | 18 | M | 217 | 9.5 | 21.21 | 0.67 | 23 | 50 | 56 |
| 3 | 18 | M | 150 | 12 | 15.04 | 0.85 | 31 | 17 | 19 |
| 4 | 17 | F | 144 | 11.3 | 13.17 | 0.8 | 32 | 17 | 20 |
| 5 | 16 | F | 220 | 8.7 | 11.9 | 0.89 | 27 | 8 | 11 |
| 6 | 18 | M | 74 | 10.4 | 20.77 | 0.6 | 27 | 9 | 12 |
| 7 | 16 | F | 97 | 7.4 | 13.3 | 0.69 | 22 | 11 | 13 |
| 8 | 22 | M | 180 | 8 | 24.32 | 2.87 | 110 | 13 | 10 |
| 9 | 20 | M | 295 | 8.5 | 15.5 | 1 | 39 | 9 | 10 |
| 10 | 22 | M | 186 | 7.8 | 14.5 | 1 | 47 | 10 | 12 |
| 11 | 20 | M | 159 | 8.3 | 18 | 0.82 | 22 | 29 | 26 |
| 12 | 22 | M | 260 | 8 | 22.18 | 0.9 | 28 | 10 | 13 |
| 13 | 20 | M | 188 | 9.2 | 17.02 | 1.4 | 27 | 9 | 12 |
| 14 | 22 | M | 150 | 8.2 | 22.14 | 0.9 | 31 | 11 | 17 |
| 15 | 21 | M | 116 | 9.4 | 17.96 | 1.1 | 32 | 17 | 20 |
| 16 | 24 | F | 168 | 12.5 | 15.44 | 3.1 | 97 | 9 | 7 |
| 17 | 22 | M | 210 | 8.2 | 18.75 | 0.72 | 9 | 11 | 12 |

**S1 Table F:** Clinical data of T1D patients with fasting blood sugar ≤160 mg/dl.

| **Patient**  **No.** | **Age (years)** | **Gender** | **Fasting Blood Sugar (mg/dl)** | **HbA1c**  **(%)** | **BMI**  **(Kg/m^2^)** | **Serum Creatinine**  **(mg%)** | **BUN**  **(mg%)** | **AST**  **(IU/L)** | **ALT**  **(IU/L)** |
| --- | --- | --- | --- | --- | --- | --- | --- | --- | --- |
| 1 | 10 | M | 154 | 7.6 | 11.08 | 0.8 | 34 | 9 | 10 |
| 2 | 12 | F | 145 | 9.3 | 13.59 | 0.7 | 7 | 9 | 10 |
| 3 | 13 | F | 152 | 10.2 | 11.05 | 0.9 | 8 | 9 | 10 |
| 4 | 20 | M | 159 | 8 | 18 | 0.82 | 22 | 29 | 26 |
| 5 | 18 | M | 150 | 12 | 15.04 | 0.85 | 31 | 17 | 19 |
| 6 | 12 | F | 118 | 7.4 | 8.16 | 0.5 | 9 | 9 | 10 |
| 7 | 17 | F | 144 | 11.3 | 13.17 | 0.8 | 32 | 17 | 20 |
| 8 | 22 | M | 150 | 8.2 | 22.14 | 0.9 | 31 | 11 | 17 |
| 9 | 21 | M | 116 | 9.4 | 17.96 | 1.1 | 32 | 17 | 20 |
| 10 | 18 | M | 74 | 10.4 | 20.77 | 0.6 | 27 | 9 | 12 |
| 11 | 13 | M | 154 | 9 | 12.82 | 1.4 | 78 | 12 | 17 |
| 12 | 11 | F | 142 | 8 | 11.7 | 0.67 | 28 | 10 | 11 |
| 13 | 7 | M | 98 | 9.3 | 10.37 | 0.54 | 43 | 9 | 12 |
| 14 | 16 | F | 97 | 7.4 | 13.3 | 0.69 | 22 | 11 | 13 |

**S1 Table G:** Clinical data of T1D patients with fasting blood sugar >160 mg/dl.

| **Patient**  **No.** | **Age (years)** | **Gender** | **Fasting**  **Blood**  **Sugar**  **(mg/dl)** | **HbA1c**  **(%)** | **BMI**  **(Kg/m^2^)** | **Serum Creatinine**  **(mg%)** | **BUN**  **(mg%)** | **AST**  **(IU/L)** | **ALT**  **(IU/L)** |
| --- | --- | --- | --- | --- | --- | --- | --- | --- | --- |
| 1 | 15 | F | 186 | 10.8 | 17.3 | 0.7 | 25 | 10 | 13 |
| 2 | 16 | M | 215 | 9.1 | 13.67 | 0.9 | 34 | 17 | 21 |
| 3 | 12 | F | 199 | 9.5 | 15.3 | 0.46 | 36 | 12 | 15 |
| 4 | 22 | M | 180 | 8 | 24.32 | 2.87 | 110 | 13 | 10 |
| 5 | 20 | M | 295 | 8.5 | 15.5 | 1 | 7 | 9 | 10 |
| 6 | 9 | M | 167 | 8.1 | 11.76 | 0.9 | 33 | 10 | 11 |
| 7 | 8 | F | 189 | 11.9 | 12.16 | 0.37 | 40 | 11 | 17 |
| 8 | 22 | M | 186 | 7.8 | 14.5 | 1 | 47 | 10 | 12 |
| 9 | 18 | M | 217 | 9.5 | 21.21 | 0.67 | 23 | 50 | 56 |
| 10 | 22 | M | 260 | 8 | 22.18 | 0.9 | 28 | 10 | 13 |
| 11 | 20 | M | 188 | 9.2 | 17.02 | 1.4 | 27 | 9 | 12 |
| 12 | 15 | F | 215 | 8.9 | 14.81 | 0.93 | 28 | 7 | 9 |
| 13 | 16 | F | 220 | 8.7 | 11.9 | 0.93 | 27 | 8 | 11 |
| 14 | 24 | F | 168 | 12.5 | 15.44 | 0.89 | 97 | 9 | 7 |
| 15 | 22 | M | 210 | 8.2 | 18.75 | 0.72 | 9 | 11 | 12 |
